# Supplementary material for: Occupational stress and environmental impact among traffic police officers in Kathmandu Valley, Nepal: A qualitative study
Source: PLOS Glob Public Health. 2023 Nov 21;3(11):e0002174. doi: 10.1371/journal.pgph.0002174 (PMC10662712; doi:10.1371/journal.pgph.0002174)
Supplement: S1 Text — (DOCX) [file pgph.0002174.s001.docx]

**Interview guideline**

**Background information**

Age: …………………………………………...

Sex: …………………………………... Rank: ………………………………………….

Years of Service: ……………………. Current Duty Position: …………………………

**General life and workload**

- Please tell us about the general life of traffic police, their living and working conditions.
- How is traffic police workload- overloaded or okay?
- Duty hours?

**Basic supplies and amenities**

- Are there problems of drinking, smoking and domestic violence among traffic police?
- Please tell us about the basic supplies and equipment: shortage, too old, modern.
- Who decides promotion and transfer of officer?

**Work life balance and Work stress**

- Do you get enough time to meet your family and friends?
- What is the impact of work-related stress on your family and friends?
- What do you consider the most stressful aspect of working as a traffic police?
- What are the effects of personal stressor on your job performance?
- What are the effects of stress experienced at work on your health and wellbeing?
- Is there any information or aspect of work-related stress that affects traffic police which you would like to share?
- Is there any provision of training to overcome or release your stress? If yes, please tell us about that.
- What are your views about counselling for the traffic police officer?

**Working environment**

- What do you think of Kathmandu’s work environment? (*Probing: Pollution, blow of horn and dusts)*
- Have you ever experienced any respiratory/skin/eye related problems?
- How can the work environment be made better for traffic police?

**Any suggestions to the concerned authorities**

- In your view, what should be done to make a friendly working environment for the traffic police?

**Anything or any stories you would like to add?**

**Thank you for your valuable time! Have a good time!**
