# Supplementary material for: Occupational stress and environmental impact among traffic police officers in Kathmandu Valley, Nepal: A qualitative study
Source: PLOS Glob Public Health. 2023 Nov 21;3(11):e0002174. doi: 10.1371/journal.pgph.0002174 (PMC10662712; doi:10.1371/journal.pgph.0002174)
Supplement: Attachment — Submitted filename: Response to reviewers 9.3.2023.docx [file pgph.0002174.s006.docx]

Reviewer #1: Dear Authors,
Your work is well put together.
These are a few things you should take a look at.

Abstract:
Lines 29-30: The six themes that you identified may perhaps be presented more distinctively, especially the "work environment and health problems".

*Response: Thank you for pointing this out. We have* modified the "work environment and health problems" into "Work environment and occupational health problems" *since we have discussed the impact of working conditions on individuals’ health condition.*

Suggestions:
1. Since you referred to "occupational health" in Line 362 of your conclusion, I suggest you modify the "work environment and health problems" into "Work environment and occupational health problems" since a work environment may not necessarily translate into health problems.

*Response: Thank you for the suggestion. We have incorporated it.*

Line 31: While I understand that your research is qualitative, I still suggest you support the work "Most" with numerical data, like how many participants reported this. A simple number or percentage will give a clearer picture.

*Response: Thank you. We have added the number.*

Introduction:
Lines 62-64: Perhaps replace this sentence "These types of hazardous roles and responsibilities place them in challenging and stressful situations that can significantly impact their mental well-being and possibly even performance" with this
“Such risky and demanding tasks expose them to difficult and tense scenarios that can affect their mental health and potentially their work quality.” for more clarity.

*Response: Thank you. We have replaced it.*

Methods:
Lines 99-102: This expression "The study of mental health among traffic police personnel in Kathmandu Valley is particularly relevant as the city's rapid urbanization has resulted in an increase in the number of vehicles on the roads and the issue of pollution is also of great concern, with the city being ranked as one of the most polluted cities in the world", may be replaced with "This study is relevant because traffic police personnel in Kathmandu Valley, which ranks among the most polluted city in the world, face unique mental health challenges as the city undergoes rapid urbanization with a growing number of vehicles on the roads and the severe pollution problem."

*Response: Thank you. We have replaced it.*

Lines 116: "Participants were recruited and interviews were carried out between the period October 2018 to..." should be corrected to "Participants recruitment and interviews were carried out between the period October 2018 to..."

*Response: Thank you. We have replaced it.*

Results:
Lines 139-140: Please refer to comments on lines 29-30 above.

*Response: Thank you. We have addressed it.*

Line 240: Check the spelling of "menstruation". It is spelt here as "mensuration", I am not sure if you wanted to leave it as pronounced by participants.

*Response: Thank you for pointing it out. We have corrected it.*

References:

Please add the DOIs or URLs of all the references listed in the document

Line 439: Reference 27 has some boxes in the lines, kindly fix them

*Thank you for pointing out our error. We have added DOIs/URLs in all references as well.*

Reviewer #2: The manuscript does not explicitly mention whether the authors have made all data underlying the findings available. As a qualitative study, data availability may vary depending on the specific research context and ethical considerations. In this particular case, qualitative data may be sensitive and require careful anonymization or protection to ensure participants' confidentiality. i think it is appropriate for the authors to provide clarity on data availability or justification for not sharing the data in this manuscript.

*Thank you for pointing it out. We have justified the data availability in the ethical considerations.*

Reviewer #3: Based on the defined scope of this study, the authors have adequately demonstrated that understanding occupational stress and environmental impact among traffic police officers can be one way to improving the quality of their work life, enhancing public safety, and creating a healthier and more resilient workforce.
The qualitative manuscript has explored the experiences and coping mechanisms of traffic police officers in a busy metropolitan area, focusing on occupational stress and its impact on their well-being. Through in-depth interviews and thematic analysis, the study has revealed the significant challenges officers face, and emphasized the need for organizational support, including mental health resources and flexible work schedules, to enhance well-being. Overall, the research sheds light on the complex interplay between occupational stress and environmental factors, offering valuable insights for policy development and fostering a healthier and more effective traffic policing environment.
You can find my minor edits in the manuscript attached.

*Thank you for the edits and comments. We have incorporated all.*

Reviewer #4: Few minor questions which the author need to answer
1. How the study is different from 'Prevalence and factors associated with symptoms of depression, anxiety and stress among traffic police officers in Kathmandu, Nepal: a cross-sectional survey'. plus there are some other similar studies carried out in the same area. give a proper justification.

*Thank you for the question. In the above-mentioned study, we quantitatively assessed depression, anxiety, stress, and associated factors among traffic police officers using standardized tools. Smoking, long working hours, and lack of physical exercise were associated with depression, anxiety, and stress. In the current paper, we have used the qualitative approach to explore the work-related factors causing occupational stress among traffic police officers. The qualitative findings were not covered in the quantitative paper. We believe that focusing on qualitative findings separately will provide deeper insight into the occupational environment and stressful factors associated with traffic police officers’ daily routine and will persuade the concerned authorities to take necessary steps towards creating a healthy working environment.*

2. How the sample size of 15 is chosen?

*Thank you for the comment. Details of sampling and sample size have been explained in the methods (participants section). We followed the principle of saturation for sample-size recruitment. We have added the following “Recruitment of participants was stopped after reaching data saturation”.*

3. there is statistical analysis shown in the manuscript.

*Thank you for the comment. There is no statistical test of significance in this manuscript. Qualitative data analysis has been explained in the data analysis section whereas Table 1 shows the demographic distribution of the selected participants.*

**Occupational stress and environmental impact among traffic police officers in Kathmandu valley, Nepal: a qualitative study**

I have carefully examined the research findings and methodology presented in this paper. My comments aim to provide constructive feedback to the authors, with the objective of enhancing clarity, significance, and overall quality of the study. I comment the authors for their valuable contributions to the understanding of occupational stress among traffic police officers in Kathmandu valley and look forward to assisting in strengthening the manuscript through thoughtful and evidence based feedback.

**Introduction:**

There are a few areas that could be improved or expanded upon. Here are some suggestions:

1. Background and Context: Provide a more comprehensive background on the occupational health challenges faced by law enforcement officers worldwide, not just in the introduction but also in the literature review section. This will help situate the study within the broader context of occupational stress among police officers. Include specific statistics or data related to the prevalence of mental health problems and the impact of occupational stress on police officers globally or in similar settings. This will strengthen the rationale for conducting the study.
2. Research Gap and Objectives: Clearly state the research gap that this study aims to address. What specific aspects of occupational stress and environmental impact among traffic police officers in Kathmandu Valley have not been adequately explored in existing literature?

Provide clear research objectives or research questions that guide the study. This will help readers understand the purpose and direction of the research.

3. Theoretical Framework: Consider incorporating a theoretical framework or conceptual model that supports the understanding of occupational stress and its impact on traffic police officers. This will provide a theoretical foundation for the study and help interpret the findings.
4. Significance and Implications: Expand on the significance of the study by discussing the potential implications of understanding work and environment-related stressful factors for traffic police officers. How can this knowledge contribute to the well-being and performance of traffic police officers? How can it inform interventions or policies to improve their occupational health?

sDiscuss the broader societal implications of the study. How can addressing the occupational stress of traffic police officers contribute to public safety, traffic management, and overall well-being in Kathmandu Valley?

If the authors address these suggestions, the introduction will provide a stronger foundation for the study and better engage the reader in understanding the significance of the research topic.

*Thank you so much for the constructive feedback. We have tried to incorporate in on the revised version of the manuscript.*

**Methods:**

The authors have provided a clear description of the participants, study design, setting, data collection and data analysis. However, there are a few suggestions to enhance clarity and comprehensiveness of this section by providing a more detailed and transparent account of the study’s methodology, allowing readers to better evaluate the study’s rigor and validity.

1. Sampling Technique: Clarify the criteria used for selecting participants through purposive sampling. Explain the rationale behind including individuals nominated by the traffic unit in charge and those who volunteered. This will help justify the selection process and address potential biases.
2. Participant Demographics: While Table 1 provides some information about the participant characteristics, consider including additional details such as educational background, years of experience as traffic police officers, and any relevant demographic information that could influence their occupational stress and environmental impact. This will provide a more comprehensive understanding of the participant profile.
3. Study Design: Elaborate on the reasons for choosing a qualitative study design and the suitability of thematic analysis for exploring the work environment and stress factors among traffic police personnel. This will help readers understand why qualitative methods were chosen over quantitative methods and how the research objectives align with the chosen approach.
4. Data Collection: Provide more information on the number of interviews conducted and whether data saturation was achieved. Data saturation refers to the point at which new information or themes stop emerging from the interviews, indicating that sufficient data has been collected.
5. Data Analysis: Expand on the process of developing the analytical framework and identifying key themes. Explain the iterative nature of thematic analysis, including how themes were refined and agreed upon among the researchers.
6. Describe any software or tools used for managing and analyzing the qualitative data, if applicable.
7. Ethical Considerations: there are a few additional elements that could be included to further strengthen the ethical considerations section:
8. Informed Consent: Provide more details about the informed consent process, such as how participants were informed about the study's purpose, procedures, potential risks and benefits, voluntary participation, and their right to withdraw at any time. Mention any measures taken to ensure that participants had a clear understanding of the study and the voluntary nature of their participation.
9. Data Protection and Confidentiality: Describe the specific measures taken to ensure the confidentiality and anonymity of the participants. Explain how the collected data was stored securely, who had access to it, and how long the data will be retained. If any identifiers were present in the data, explain how they were anonymized or pseudonymized to protect participant identities.
10. Potential Risks and Benefits: Discuss any potential risks or discomfort that participants might have experienced during the interviews or due to the sensitive nature of the topic. Also, mention any measures taken to minimize these risks and provide support or referral services to participants, if applicable.

Highlight the potential benefits of the study, both for the participants (e.g., increased awareness of occupational stress, potential interventions) and for society (e.g., improved working conditions for traffic police officers).

By incorporating these additional elements, the ethical considerations section will provide a more comprehensive overview of the steps taken to protect the rights and well-being of the study participants.

*Thank you for the opportunity to strengthen our work. In the revised version we have tried to address your concerns.*

*Participant selection was based on nomination done by the traffic unit in charge and by volunteerism that included the individuals who were deemed capable of providing the most comprehensive and informative data. This has been described in the methods section of the manuscript.*

*Information regarding data saturation has been added in the ‘participants’ section of the manuscript. Similarly, rationale for using qualitative approaches to understand occupational stress among the traffic police has been added.*

**Results:**

The authors have presented the findings of the qualitative study well, highlighting six major themes: workload, work-life balance, basic amenities, work environment and health problems, and possible solutions and suggestions. The findings provide valuable insights into the experiences and perspectives of traffic police officers in Kathmandu Valley. However, here are a few suggestions to improve the presentation of the results:

1. Clarity in Theme Presentation: Consider organizing the presentation of themes in a more structured and explicit manner. Provide clear subheadings for each theme and use consistent formatting throughout the section. This will enhance the readability and help readers navigate through the findings more easily.
2. Richness of Findings: To provide a richer understanding of the participants' experiences, consider including more direct quotes or examples from the interviews. Including verbatim quotations will make the findings more vivid and allow readers to connect with the voices of the participants.

Provide a range of perspectives within each theme. It would be beneficial to include a diversity of participant quotes to demonstrate the variation and depth of experiences related to each theme.

3. Interpretation and Discussion: After presenting each theme, consider adding a brief interpretation or discussion paragraph. This can involve explaining the significance of the findings, linking them back to the existing literature, and discussing their implications for the well-being of traffic police officers and potential interventions.
4. Validity and Trustworthiness: Briefly discuss the steps taken to ensure the validity and trustworthiness of the findings, such as member checking (where participants review the findings) or researcher reflexivity (acknowledging the potential influence of researchers' biases). This will enhance the transparency and rigor of the study.

The authors should consider incorporating these suggestions so that this section does provide a clearer, more detailed, and engaging account of the findings, enabling readers to grasp the nuances of the traffic police officers’ experiences and the implications of the study.

*Thank you for the suggestions. We have worked accordingly.*

**Discussion:**

This section provides a comprehensive overview and analysis of the study's findings, linking them to existing literature and drawing meaningful conclusions. The authors highlight the major themes that emerged from the interviews and discuss their implications for the mental well-being and job performance of traffic police officers in Kathmandu Valley. The discussion also includes strengths and limitations of the study and concludes with valuable suggestions for policymakers and stakeholders.

However, here are a few suggestions to further enhance the discussion:

1. Linkage to Existing Literature: While the discussion section mentions some studies conducted among police officers in different contexts, consider expanding the discussion to include a more comprehensive review of the literature on occupational stress and mental health among law enforcement personnel, including traffic police officers, globally. This will provide a broader context for understanding the significance of the study's findings.
2. Interpretation of Findings: Provide deeper interpretations of the study's findings and link them back to the study's objectives and research questions. Discuss the implications of the identified themes for the mental health and well-being of traffic police officers, and how these stressors can impact their job performance and overall occupational satisfaction.
3. Practical Implications and Recommendations: Elaborate on the practical implications of the study's findings for traffic police officers' well-being and job performance. Discuss how the identified stressors can be addressed and provide concrete recommendations for policymakers and police departments to improve the working conditions and mental health support for traffic police officers. Consider addressing the financial and infrastructural aspects that may be needed to implement these recommendations.
4. Generalizability and Transferability: Acknowledge the limitations of the study in terms of its generalizability to other regions and countries. Discuss the potential transferability of the findings and how they may inform similar studies in different contexts.
5. Future Research Directions: Suggest potential avenues for future research based on the study's findings. Consider areas where more in-depth investigations are needed to understand specific aspects of traffic police officers' occupational stress and well-being.

By incorporating these suggestions, the discussion section will become more comprehensive and provide a well-rounded analysis of the study's findings, leading to more informed and actionable conclusions and recommendations.

*Thank you. We have incorporated the above suggestions.*

**Abstract:**

Here are some suggestions for what could be added to the abstract:

Highlight the Importance of the Study: Include a sentence in the introduction or conclusion of the abstract that emphasizes the significance of studying occupational stress among traffic police officers. Mention the potential implications for public safety, the well-being of law enforcement personnel, and the overall functioning of traffic management in the Kathmandu Valley.

Address Specific Work Environment Stressors: The abstract could provide more specific examples of the hazardous working conditions and challenges faced by traffic police officers in Kathmandu Valley. For instance, mention the impact of exposure to vehicular emissions, noise pollution, and road congestion on their mental well-being.

Discuss Recommendations and Solutions: While the abstract mentions "possible solutions and suggestions," it could provide more specific details on the recommendations proposed by the participants. Mentioning specific strategies suggested by the traffic police officers, such as traffic rule awareness campaigns, improved infrastructure, and increased leave and duty opportunities, would add depth to the abstract.

Mention Study Limitations: Briefly state any limitations of the study. For example, mention the specific context of Kathmandu Valley and how the findings may not be directly generalizable to other regions or countries with different traffic management systems and working conditions.

Emphasize Call-to-Action: The abstract could conclude with a strong call-to-action, urging policymakers, organizational leaders, and stakeholders to take proactive steps in implementing the suggested solutions and prioritizing the mental well-being and safety of traffic police officers.

By incorporating these elements, the abstract will provide a comprehensive overview of the study's objectives, methods, findings, and implications, encouraging readers to explore the full paper for a deeper understanding of the research.

*Thank you for the opportunity to strengthen our manuscript. We have revised accordingly.*
